# Endurance training-induced changes in heart rate and blood lactate concentration in puppies

**DOI:** 10.3389/fvets.2026.1770294

**Published:** 2026-03-31

**Authors:** Heli Hyytiäinen, Jouni Junnila, Outi Laitinen-Vapaavuori, Heikki Kyröläinen

**Affiliations:** 1 Department of Clinical Equine and Small Animal Medicine, Faculty of Veterinary Medicine, University of Helsinki, Finland; 2 EstiMates Oy, Turku, Finland; 3 NeuroMuscular Research Center, Faculty of Sport and Health Sciences, University of Jyväskylä, Jyväskylä, Finland

**Keywords:** aerobic capacity, dog, exercise, juvenile, Labrador retriever, physiotherapy

## Abstract

**Introduction:**

Dogs are used for various physically demanding sports and work activities, with training starting at a very young age. However, little is known about the effects of training on young puppies. This study investigated effects of a structured endurance program on changes in heart rate (HR) and blood lactate (BL) concentrations measured after a fitness field test in Labrador retriever puppies aged under six months.

**Methods:**

Fifteen puppies were included: eight in the training group (TRA) and seven in the control group (C). Training group puppies followed an eight-week training program, while C puppies lived their normal lives without any intervention. The amount and intensity of locomotion were monitored continuously. Changes in HR and BL were measured after field tests consisting of a 1,000 m run and a 200 m sprint, followed by a recovery time until HR returned to baseline. Measurements were taken at baseline, mid-timepoint (four weeks of training), end of training (eight weeks of training), and after a detraining period (four weeks after end of training).

**Results:**

No adverse short-term effects were observed in relation to the training program. Most physiological outcomes were non-significant. After the detraining period, in the TRA-group, HRs were 32 beats per minute lower (*p* = 0.040) after the 200 m sprint than at baseline. The only significant change (*p* = 0.028) was observed in the BL concentration after the 200 m sprint; after the detraining period, it was lower than after the end-timepoint in the TRA-group.

**Discussion:**

A safe, periodized training program of varying volume, intensity, and frequency of physical loading was introduced. Limited significant physiological responses were, however, reported. Large variations in HR and BL levels were noted between the puppies throughout the study period. Study's imitations include small sample size, lack of randomization in group allocation, and lack of assessment of long-term impact to the musculoskeletal system.

## Introduction

The Labrador retriever is one of the most common dogs breeds^1^,^2^. They serve as companions as well as working animals. In their original purpose as retrieving hunting dogs, they are trained to perform in challenging environmental conditions for extended periods of time, requiring good aerobic capacity. However, there are no scientific publications available on how these dogs are physically trained for their work. Presumably, the dogs' trainers use their own, unstandardized training programs based on personal experiences and opinions. Labrador retrievers trained by a professional trainer have been reported to show signs of hyperthermia, respiratory alkalosis, hypocapnia, and mild metabolic acidosis after five minutes of retrieving in warm ambient temperature (29–30 °C) (1). This highlights the welfare challenges related to these dogs' work and the need for knowledge about how to appropriately train them for work.

Good aerobic capacity enables one to carry out physical activity for a prolonged period at a submaximal level. To achieve optimal training responses, training must occur regularly, be performed at least at a moderate intensity (about 50% of maximum capacity), and be of sufficient duration (2–4). In young humans, maximal oxygen uptake was improved most through 40–60-min training sessions with a training frequency of at least 3–4 times weekly (5). In addition to terrestrial training, training in water can be utilized for endurance training. Due to the lift and hydrostatic pressure caused by water, exercises can be performed at a lower heart rate (HR) on an underwater treadmill (UWTM) to achieve the same training load effects on the cardiovascular system (6). Additionally, flow of water, its higher density than air, and buoyancy reduce stress on joints (7). As training in water decreases strains on the skeletal system while allowing the cardiorespiratory system to be stressed, swimming has been promoted as a part of, for instance, young horses' training, increasing performance abilities with less musculoskeletal injuries (8). Thus, water-based training seems to be a beneficial form of training. Moreover, for working Labrador retrievers in training and competition situations, retrieving from water results in lower blood acidity (pH) than retrieving from ground (1). Given the breed-typical work element, water-based training could be considered as functional for Labrador retrievers.

As in humans, systematic training in dogs is known to have a positive effect on the function of the heart and circulatory system (9). For example, training lowers dogs' resting HR (10, 11). In a previously published study, sedentary adult Labrador retrievers were trained on a treadmill for 25 minutes three times a week for four months, at an HR intensity of 30%−50% of predicted HR max (12). The dogs' mean HR at control running speed decreased significantly, and the running speed required to maintain the control HR increased significantly after both two and four months of training. In addition to the HR changes, endurance-trained dogs can maintain their aerobic energy production better than their untrained counterparts during exercise (13).

Currently, based on our observations, the training of dogs for sport and work purposes seems to start at a relatively young age. Although little is known of the effects of endurance training in young growing dogs (i.e., puppies), aerobic capacity can be improved with optimal endurance training at all ages in humans (14). Two studies report endurance training to affect blood values, hormone levels, articular cartilage composition, and bone density in puppies (15, 16). Nevertheless, there is not only a general lack of information regarding the effect of physical training on puppies, but even age-specific reference values for physiological normal have not been reported. It is important to note that the hematologic and plasma biochemical values change between puppyhood, especially during the first year of life, and adulthood. They also differ between breeds, e.g., between Labrador retrievers and Beagles or Miniature schnauzers (17, 18). Moreover, lactate levels in ten-year-old beagles have been shown to be higher than those of two-year-old beagles on a treadmill-based endurance test. By contrast, HRs in older dogs do not elevate significantly, like they do in younger dogs (19), further supporting the need for age-related physiological values. Bearing this in mind, we have previously developed a field test specifically for use in young puppies (20). The main outcome measures in this fitness test are blood lactate (BL) concentration and HR during exercise, which are common and widely used parameters when studying dogs' endurance capabilities (12, 19, 21).

The aim of this study was to evaluate the effects of a structured endurance program on changes in HR and BL concentration measured during a fitness field test in Labrador retrievers aged under six months. In addition, we aimed to provide growth-related reference values for HR and BL concentration for these dogs. Our hypothesis was that a training program would result in significantly lower HRs and BL concentrations and faster recovery times from the fitness test at all measurement timepoints. From a practical point of view, our objective was to provide guidelines for safe and effective endurance training for young Labrador retrievers.

## Materials and methods

The Regional State Administrative Agency for Southern Finland approved the study protocol (ESAVI-11887-201). Owners of participating dogs signed an informed consent.

### Animals

Dogs were recruited by advertising the study in various relevant social media (Facebook) groups and Labrador retriever club journals. All puppies that met the inclusion criteria and whose owners registered during the predetermined data collection period were included in the study. The inclusion criteria comprised healthy Labrador retrievers aged 16 (±2 days) weeks at the start of their study period. Moreover, puppies' parents had to have a hip score of A/A and an elbow score of 0/0 recorded in the Finnish Kennel Club open database, indicating excellent hip and elbow joint health (22, 23). Exclusion criteria were any abnormality in physical examination or any signs of pain or skeletal abnormalities in orthopedic examination performed by a veterinarian at the start of the study period. After enrollment, puppies would also be excluded during fitness testing if any signs of pain, cyanosis, or other similar concerns were observed by the veterinarian performing the fitness testing or the owner. Moreover, if any unresolved health concerns were raised by the physiotherapist or the owners during the study period and training of the dog, the puppy was excluded.

**Figure 1:**
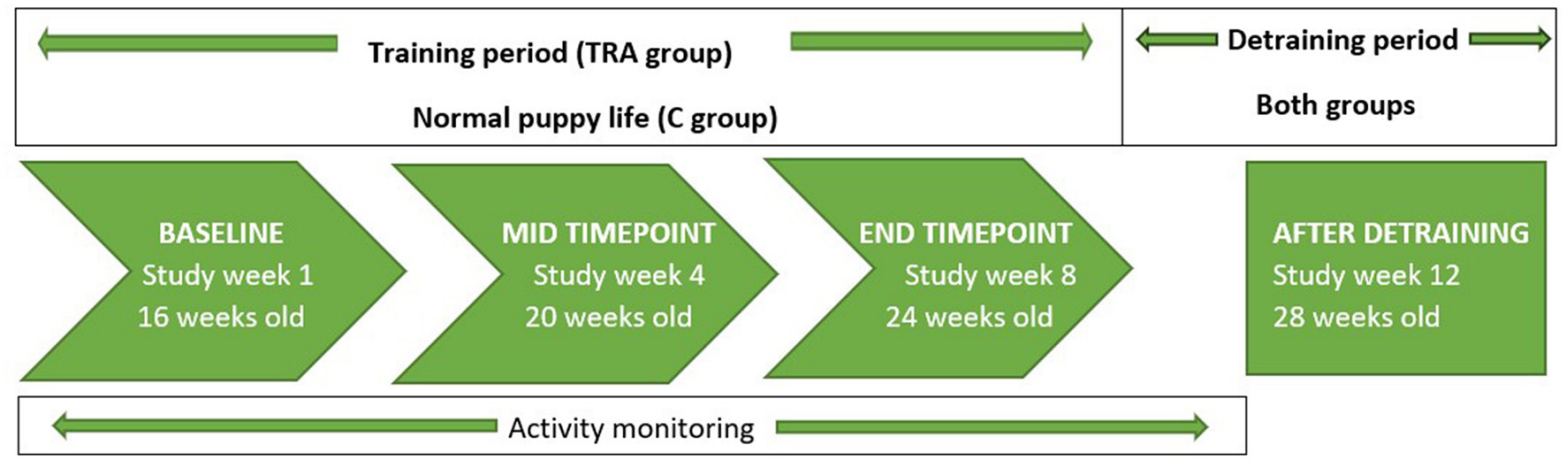
Structure and timing of the study. TRA, training group; C, control group.

Once enrolled, the puppies were divided into two groups: training (TRA) group and control (C) groups. The non-random allocation of the puppies to the groups was based on the owner's ability to commit to the two-month exercise program. The study groups and dropouts are presented in Table 1. Eight of the 15 participating puppies were siblings and were divided between the two groups, thus allowing for a genetic match between 50% of the two groups' dogs. The study protocol is presented in Figure 1.

**Table 1:** Presentation of study and control groups.

	Training group		Control group	
	Male	Female	Male	Female
Baseline *n* = 15	2	6	3	4
Excluded at mid-timepoint	–	1^*)^	–	–
Excluded prior to or at end of timepoint	–	1^*)^	–	1^**)^
Excluded prior to recovery timepoint	–	–	1^***)^	–

Reasons for exclusion: ^*^) lameness in fitness test, ^**^) lost to follow-up, ^***^) euthanasia for reason unrelated to the study.

### Physical examination

Physical examination included assessment of general appearance (perky, normal, tame, apathetic) and body condition score on a scale 1–5 (1 = cachectic, 2 = underweight, 3 = ideal weight, 4 = overweight, 5 = obese), auscultation of heart for HR, rhythm and possible murmurs, auscultation of lungs and evaluating breathing sounds (normal, highlighted, crackles, wheezing), and type of respiration (normal, panting, dyspnea, tachypnea). The color and moisture of mucosal membranes as well as capillary refill time was evaluated. Metatarsal pulse was palpated and evaluated as strong, moderate, weak, or thready. Lymph nodes were palpated for enlargement.

### Orthopedic examination

In the orthopedic examination, the puppy's movement was assessed visually and scaled 0–5 (0 = normal, no lameness, 1 = mild lameness or minor gait abnormality, 2 = moderate lameness or obvious gait abnormality, 3 = severe weight-bearing lameness, 4 = non-weight-bearing, 5 = gait cannot be evaluated). Proprioception assessment and range of motion of the joints were evaluated for each limb, and muscles were palpated for symmetry and bones for pain. Spinal vertebrae and lumbosacral space were palpated for signs of pain. All abnormal findings were recorded.

### Exercise program

The training group puppies received an eight-week endurance training program, which included a home exercise program (Supplement 1) and twice weekly training on UWT at the University of Helsinki Veterinary Teaching Hospital's Physiotherapy Department. The home training program included a progressive volume and intensity of controlled exercise, with one rest day per week. Volume of exercise was distributed to three lead walks per day. The lengths of walks varied from 1 h 20 min at the beginning of the program to 2 h 20 min at week three. Running sprints (50–200 m) were added to the program for weeks five to seven. Lighter workload was scheduled for weeks four and eight, when the fitness tests were performed (Supplement 1).

UWT training was done in ca. 28 °C warm water, with the owner always present at the session, and UWT (Keiper Water-Walker^®^, Ludwig Keiper Gmbh & Co. KG, Obermoschel, Germany) was controlled by an experienced veterinary physiotherapist. On UWT, puppies walked at individually varying distances and speeds in hip-depth water. The intensity and duration of exercise depended on the puppy's performance on UWT. The physiotherapist and the owner who was always present at the UWT training session evaluated the puppy's behavior for any signs of fatigue, which was the endpoint of each session. Signs of fatigue included excessive panting, slowing down, and lack of concentration and direction on the treadmill. At the first visit, the puppies were taught to exercise calmly and stress-free during UWT. The training was mainly done in walk, and progression to exercise was achieved through an increase in speed and duration during UWT from one session to another. Control puppies lived a normal life without restrictions or added exercise-related instructions.

**Figure 2:**
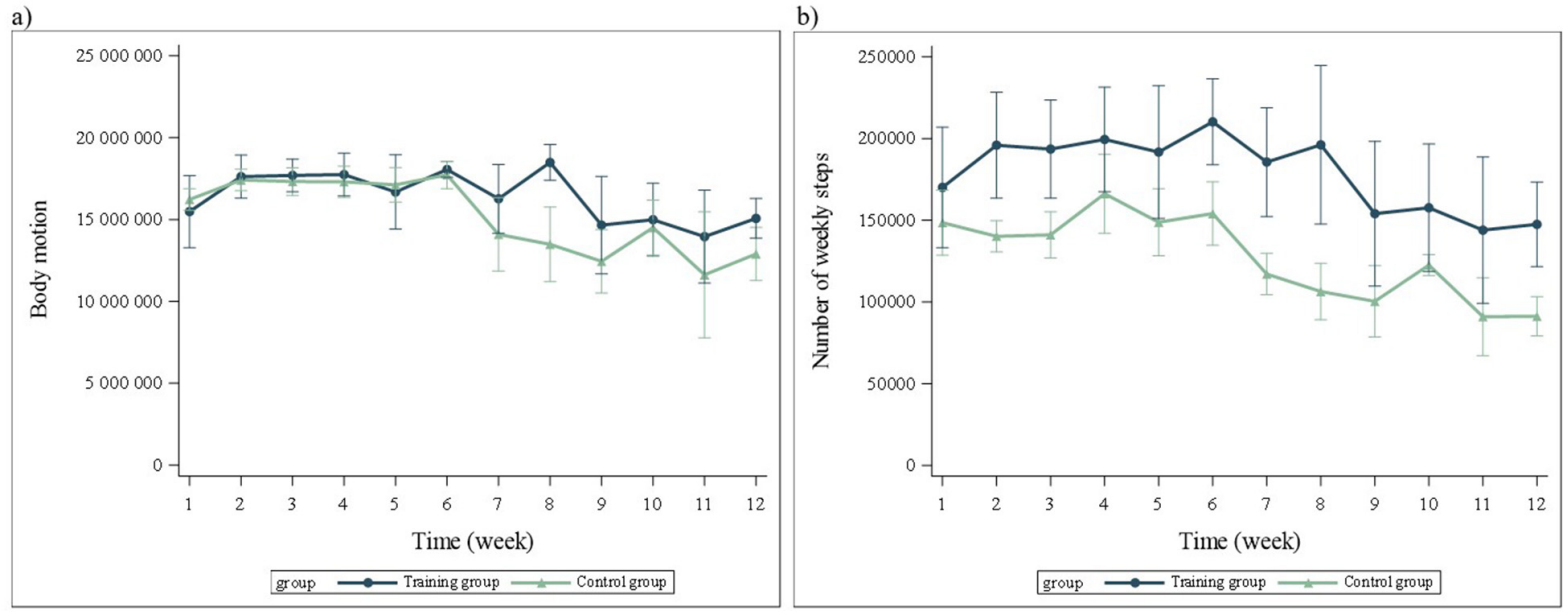
Puppies' mean (±SE) weekly physical activity **(a)** and number of steps **(b)** in both training (TRA) and control (C) groups over the two-month study period. Missing days are extrapolated. Weeks 1-8 are included in the statistical analysis; weeks 9 and 10 are merely descriptive.

To verify the amount of daily locomotion between the TRA- and C-groups, an activity monitor was used to measure the number of daily steps and the intensity of daily exercise for puppies in both groups. The activity monitor (Fuzztale, Venture Laborist LTD, Wirral, UK) was a four-axis gyroscope capturing and recording motion data continually, but an aggregate measurement was taken every five minutes and stored as a number of steps and an intensity of physical activity. This was a small plastic device weighing 25 g and measuring 4 cm in diameter that was attached to the puppies' collars throughout the study period. It was secured to the collar with a self-adhesive bandage (Coban, 3M Health care, Suomen 3M Oy, Espoo, Finland). Owners had a Fuzztale Fitness app (version 1.2.2) on their phones for the activity monitor, which they synchronized with the activity monitor every three days. Raw data were retrieved from the app by one of the authors (ES) at the second, third, and fourth fitness testing sessions (Figure 2).

### Fitness testing

Puppies in both groups were tested for their aerobic capacity four times during the three-month experimental period which included eight weeks training period followed by four week de-training period. The tests were done at the age of 16 weeks ± 2 days (baseline), 20 weeks ± 2 days, 24 weeks ± 2 days (post-training effect), and 28 weeks ± 2 days (one month after ending the training program to measure the detraining effect) (Figure 1). The fitness tests for the puppies were performed in a 500 m^2^ indoor hall with rubber-filled artificial turf (Agility Softex, Lappset Sport, Lappset Group Oy, Helsinki, Finland). The test comprised a 1,000 m run, followed by a 200 m sprint, with measurements of HR and BL concentration prior to the test, between the two distances, immediately after the test, and after a 5–8 min recovery period (20).

### Statistical analysis

Descriptive statistics were calculated for weight, UWT training-related information, the measured HRs (pre-training, after 1,000 m test, and after 200 m test), recovery time, and BL measurements (pre-training, after 1,000 m test, after 200 m test, and after recovery time) by group (TRA/C) and visit. Changes from baseline were calculated as well. In addition, changes from pre-training value (both HR and BL) after 1,000 m and 200 m tests were presented descriptively by group and visit. Changes in the BL concentration induced by the 200 m test were compared at each measured timepoint. Changes from baseline in HR were analyzed with linear mixed effects models, where the corresponding HR at baseline was used as a covariate and group, visit, and interaction between group and visit were used as fixed terms. The dog was used as a random effect in the models. Similar methodology was used to analyze recovery time, weight, and BL (at pre-training, after 1,000 m test, after 200 m test, and after recovery). In addition, the change from BL after recovery from the 200 m test was analyzed with a similar model to evaluate the decrease of lactate concentration directly after the tests. Descriptive statistics for the number of steps and body motion were calculated by treatment group and week. The treatment means over time were illustrated graphically, as were curves for individual dogs. As some dogs had days that the number of steps/body motion had not been successfully recorded in the software, the missing days were extrapolated by using the average number of steps/body motion of the corresponding week for the dog. Both original values and extrapolated values are presented in the tables.

Number of steps and body motion during the eight-week training period were both analyzed with the linear mixed effects models, where treatment group, week, and interaction between treatment group and week were used as fixed terms, dog as the random effect, and compound symmetry as the covariance structure. The models were constructed for both the original values and the values with the extrapolated missing days. Results based on the extrapolated data are presented. All statistical calculations were performed using SAS software version 9.4 (SAS Institute Inc., Cary, NC, USA). *P*-values < 0.05 were considered statistically significant.

## Results

At baseline, the mean (SD) body masses of the puppies in the TRA and C groups were 13.2 (1.64) kg and 13.0 (2.12) kg, respectively. The TRA-group puppies gained over seven kilograms of body mass during the study period, whereas the C-group puppies gained over eight kilograms (Table 2). No significant difference emerged in body mass between the groups at four, eight, or twelve weeks (*p* = 0.913, *p* = 0.273, and *p* = 0.292, respectively).

**Table 2:** Outcome measurement results at various timepoints per group.

Timepoint	Puppies' age (weeks)	N	Body mass (kg)	Lactate pre test (mmol/l)	Lactate after 1,000 m run (mmol/l)	Lactate after 200 m sprint (mmol/l)	Lactate after recovery (mmol/l)	Heart rate pre-test (bpm)	Heart rate after 1,000 m run (bpm)	Heart rate after 200 m sprint (bpm)
Training group										
Baseline	16	8	13.2 (1.6)	0.9 (0.5)	1.0 (0.5)	1.2 (0.6)	1.2 (0.5)	146 (19)	173 (38)	171 (25)^*^
4th week	20	7	16.8 (1.8)	1.3 (0.8)	1.6 (0.7)	1.6 (0.7)	1.4 (0.8)	131 (7)	164 (13)	162 (26)
8th week	24	7	19.4 (2.9)	0.7 (0.5)	1.5 (0.5)	1.7 (0.6)	0.9 (0.4)	122 (15)	162 (24)	175 (29)
12th week	28	6	21.4 (3.1)	1.1 (0.5)	1.0 (0.6)	1.0 (0.7)	1.1 (0.5)	117 (12)	137 (31)	141 (43)^*^
Control group										
Baseline	16	7	13.0 (2.1)	0.9 (0.5)	1.0 (0.8)	1.0 (0.6)	1.0 (0.4)	139 (21)	155 (31)	172 (27)
4th week	20	7	16.9 (2.1)	0.9 (0.4)	1.3 (0.8)	1.4 (1.0)	0.9 (0.4)	129 (17)	163 (48)	172 (40)
8th week	24	6	20.4 (2.1)	0.7 (0.2)	0.9 (0.5)	1.2 (0.7)	1.3 (0.9)	125 (17)	159 (33)	173 (32)
12th week	28	5	22.3 (2.3)	0.9 (0.9)	0.3 (1.6)	0.9 (0.7)	1.1 (0.6)	120 (13)	162 (45)	164 (36)

Mean (±SD) changes in body mass, lactate concentration, and heart rate values for untrained and trained puppies in a fitness test. Significant change to baseline marked with an asterisk. N, number of dogs; mmol/l, millimoles per liter; bpm, beats per minute.

**Figure 3:**
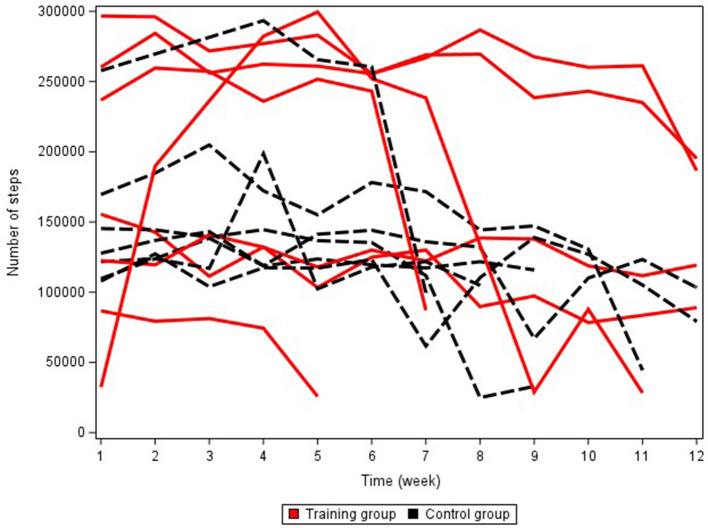
Individual number of steps-time curves in the training (TRA) and control (C) groups. Missing days are extrapolated.

Physical activity of the puppies over the training period did not differ significantly (*p* = 0.697) between the two groups (Figure 2). The step count between the groups also did not differ significantly (*p* = 0.255) over time. However, there was a trend of the TRA-group being more active than the C-group, with a clearly higher weekly number of steps (64,282 more steps) than the C-group puppies at the eighth week of the study period (Figure 3). At eight weeks, the extrapolated number of weekly steps was 196 150 (96 981) for the TRA-group's puppies and 106 391 (42 408) for the C-group's puppies, with no significant difference between the groups (*p* = 0.137). The step count of one TRA puppy was clearly different from the rest, and this puppy was excluded at the start of the last fitness test due to lameness (the lowest and shortest curve in Figure 3).

According to the exercise program (Supplement 1), the TRA-groups' puppies were planned to have 15 UWTM sessions: twice a week except only once in week four when fitness test was done. Four puppies participated in all scheduled training sessions. One puppy missed three training sessions and two puppies missed one. Puppies trained 17–27 minutes per session during the study period. The speed of UWTM ranged from 6.5 meters per minute to 32.5 meters per minute, and the duration of exercise ranged from 6 to 48 min between training sessions. The puppies' mean HR ranged from 115 beats per minute (bpm) to 200 bpm (mean 154 bpm), with most of the HR values staying below 180 bpm throughout the UWTM training sessions.

**Figure 4:**
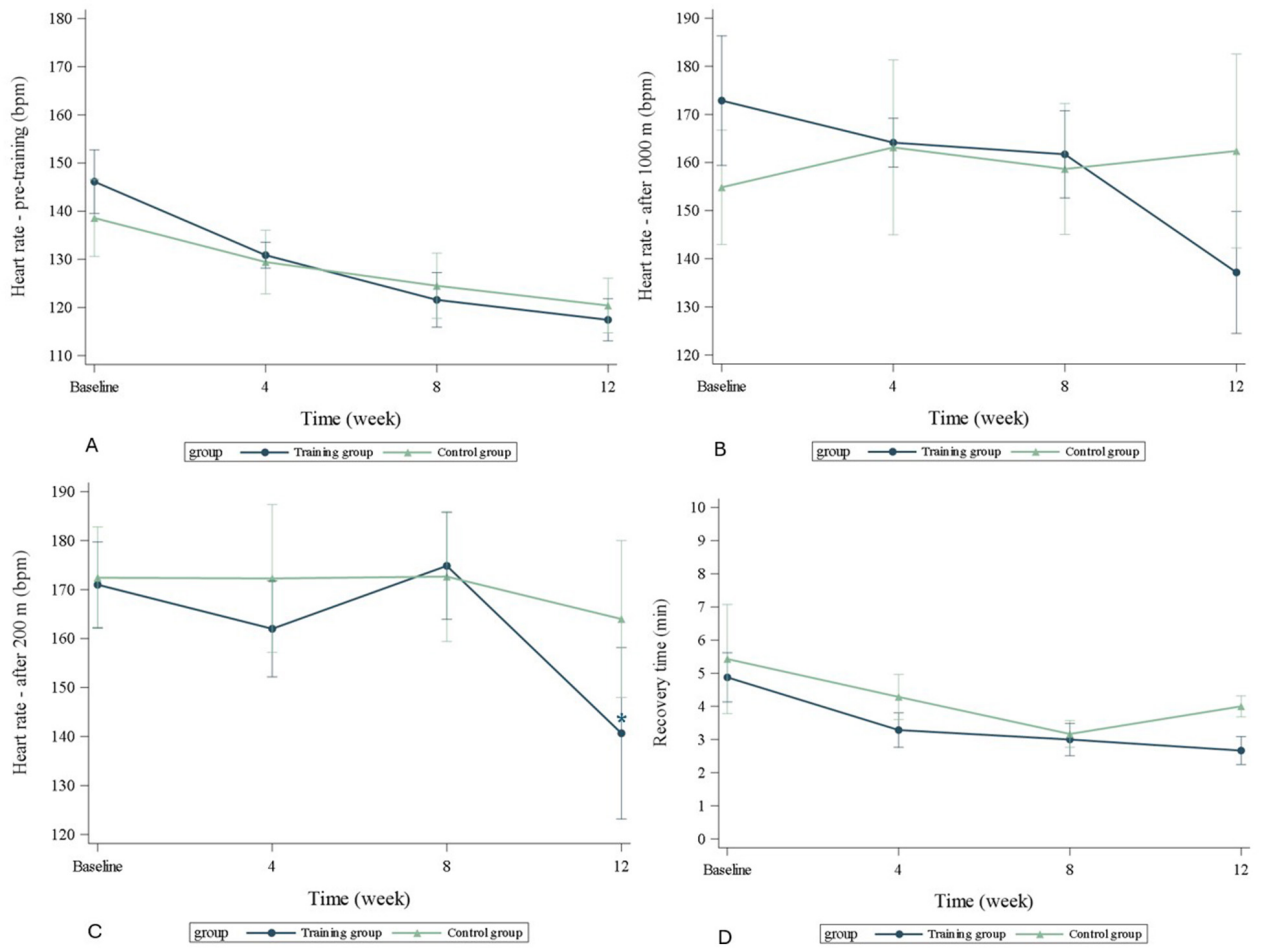
Changes in **(A)** baseline heart rate, **(B)** heart rate after 1,000 m run, **(C)** heart rate after 200 m sprint, **(D)** heart rate at recovery. Signifigant change (no change in absolute values) within the group is marked with and asterix. TRA, training group; C, control group.

**Figure 5:**
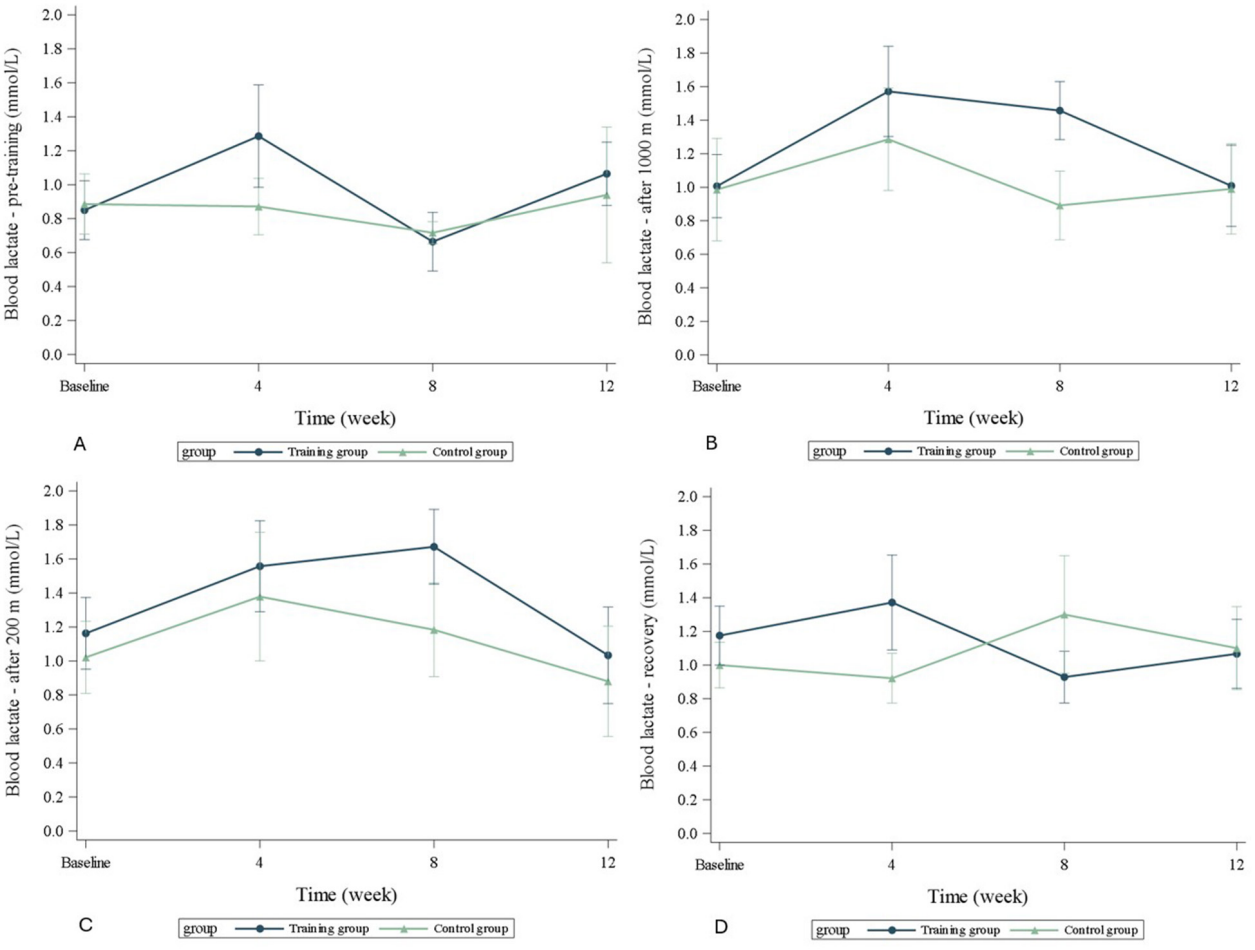
Changes in **(A)** baseline blood lactate level, **(B)** blood lactate level after 1,000 m run, **(C)** blood lactate level after 200 m sprint, **(D)** blood lactate level at recovery. TRA, training group; C, control group.

None of the HR or BL values were significantly different between the TRA- and C-groups at any studied timepoint (Table 2, Figures 4, 5). In the TRA-group, the change in HR after the 200 m sprint differed significantly (*p* = 0.040) between baseline and after 12 weeks of training, with an estimated 32 beats per minute less. In the BL analysis, the only significant change (*p* = 0.028) was observed in the BL concentration between the 200 m sprint and the recovery lactate values in the TRA-group at week eight. None of the other measured changes within the groups from baseline were significant.

**Figure 6:**
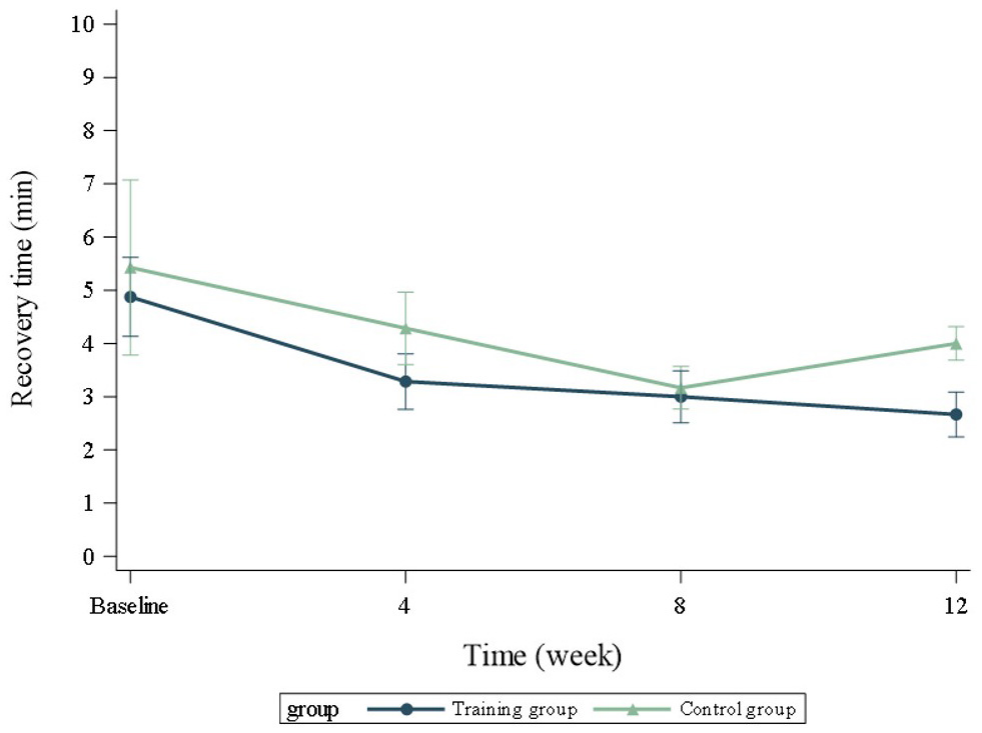
Changes in recovery time in minutes over time after the 1,000 m run and 200 m sprint. TRA, training group; C, control group.

Recovery time between the TRA- and C-groups did not differ significantly at any timepoint. The recovery time decreased in both groups at each measurement time, except for the C-group's last measurement (Figure 6). For the TRA-group, the recovery time shortened significantly from baseline to the test points at four, eight, and twelve weeks (*p* = 0.003, *p* < 0.0001, and *p* < 0.0001, respectively). For the C-group, the changes from baseline were quite similar and also significant for all three measurement points (*p* = 0.030 at week four, *p* < 0.001 at week eight, and *p* = 0.019 at week twelve). After the whole training period (at week eight), the estimated decrease in recovery time for the TRA-group was−2.32 min (lower 95% CI−3.27 min, upper−1.36 min) and for the C-group−2.20 min (lower−3.23 min, upper−1.17 min).

## Discussion

This study investigated the effect of a structured 12-week endurance program (with eight week training and four week de-training periods) on HR and BL concentration levels after exercise among young, client-owned Labrador retriever puppies. The primary finding was that HR values after the 200 m sprint were significantly lower after the detraining period than at baseline. The reason for only this timepoint being sensitive to change is not known to us, but it could be speculated that they might be due to the overall loading (other activities added to training program and accumulation of stress) being too large. Therefore, after the detraining period, positive changes were observed, i.e., the puppies were able to run more economically, with lower HR. This is supported by significantly higher BL concentration after the 200 m sprint at the eighth training week than at baseline. The true impact of training might have been more visible later than the four weeks detraining. As seen also in our results, there is a great individuality in adaptation to training. The overall load of the training period cumulates from the training as well as from all other activities/stresses of everyday life. This leads to individual differences and possibly to the effect not showing before the detraining. The individual differences combined with the low number of study animals may have affected the results.

No adverse effects related to the training or fitness testing were observed among the puppies, demonstrating that our training program was safe. This is important information in itself. On the other hand, no significant differences were detected in the parameters of the fitness test between trained and untrained puppies. Thus, our training program may not have been sufficiently effective to cause physical and physiological changes. Instead, it was noted based on the HR that the UWT training was not performed at as high a stress level as it could have been. Avoiding any detrimental impact on the developing neuromusculoskeletal system was also a priority in our study. However, a few previously published studies reported that several hours of daily repeated relatively large amounts of training would not significantly impact some growth factors of Beagle puppies. Their growth plates closed normally, and the bones of the limbs actually became stronger than in the control group (15, 16, 24, 25). However, slight negative effects were also reported in relation to the bone density of the puppies' spines, the composition of the articular cartilage, blood values, and hormonal regulation (15, 16, 24, 25). Thus, due to the limited amount of information and the fact that the animals used in this study were client-owned young puppies, all interventions erred on the side of caution, with safety as a primary requirement. However, parameters related to possible long-term joint or musculoskeletal damage due to training were not included nor controlled as part of this study. The possible long-term impacts (or their definite exclusion) should be considered in future studies. Another specific concern was exercise-induced collapse, a condition to which Labrador retrievers are susceptible (26). This condition has been reported to affect young adult dogs (26, 27), but as there is no literature on puppies being exercised strenuously and purposefully, we did not know how young dogs might manifest signs if pushed too far. Collapse episodes may be brought on by overly strenuous exercise, and as we were not able to define and quantify “strenuous” for each puppy, high loads of physical stress were avoided. However, more research on strain- and training-related guidelines of endurance-type physical training in growing dogs, specifically breeds with exercise-related diseases, is needed. So, although we did not cause harm, we also did not achieve the desired results. Despite partly disappointing results, the data are important for future studies, where, based on this information, more strenuous programs could be applied.

Being unaware of the maximum HR (HRmax) of the puppies in our study led into rather subjective assessment of an appropriate level of training for each puppy. In humans, endurance training is traditionally divided into three HR categories: basic, speed, and maximal endurance (28). On this scale, the upper limit for basic endurance training is about 80% and for speed endurance training 90% of the maximal HR (28). At 80% and 90% HRmax, lactate levels start to increase and to accumulate in the body, respectively. There is no literature available on the HRmax for young puppies, and due to safety precautions, we could not test the true HRmax in these puppies. Based on the previous literature, mature dogs have been reported to have slightly over 300 bpm HRmax (29–32). Since aging decreases dogs' HRmax (33), one could assume that the puppies' HRmax is at least as high as that of adults. With this reasoning, basic training should have been done at 60%−80% HRmax (180–240 bpm), speed endurance at 80%−90% HRmax (240–270 bpm), and maximal endurance training at 90%−95% HRmax (270–285 bpm). Our puppies' training HRs at UWT were not at these levels, with most of the training sessions being performed below 180 bpm (60% HRmax). Even during the fitness testing at week eight after the 200 m sprint, the mean HR was 175 bpm; thus, only 58% HRmax. The above reasoning is based on human HR thresholds and may not actually be applicable to dogs, who have wider range of HR, and their basic endurance training thresholds may cover much lower HR values. In humans, due to the lift and hydrostatic pressure caused by water, during UWT it has been noted that one can move on average at seven beats lower HR than on a dry treadmill to achieve the same load effects on the heart and respiratory system (6). The lower HR is enabled by the pressure of the water increasing venous return, resulting in an increase in the heart stroke volume (34). Probably also in dogs, HR during water therapy will not rise as high as during dry land exercise, even when the respiratory and circulatory systems are subjected to an equal load. Hence, although the HR values for UWT training did not reach the calculated values for endurance training, they may still have been at an optimal or near-optimal training level. This phenomenon warrants further studies to enable effective use of UWT for sports dogs' training and possibly also as a safe mode to increase training load on patients whose heart condition does not allow strenuous training on dry land.

To optimize the standardization and control of the levels of activity between groups, activity measurements in our study were taken to show differences in weekly physical activity levels between the TRA- and C-groups. Although not statistically significant, a clinically significant difference in the number of steps was seen; the training group performed more overall physical activity. In addition, the result regarding the difference in step counts between the groups is most likely affected by the number of animals. Had we had more dogs in our groups, the difference between them might have been significant, as the difference between groups was already 64,000 steps per week, which can be considered a clinically noteworthy difference. Despite a difference in step numbers, physical fitness did not differ between the groups when measured with the selected methods and parameters. This lack of difference can stem from the above-discussed evaluation that the instructed amount and intensity of exercise was too low to result in a measurable impact on the puppies compared with their non-trained counterparts. On the other hand, it can also be that the Labrador retriever puppies' owners exercise their puppies at such an endurance training level intuitively, i.e., that all puppies are already “athletic” rather than just “normal untrained” ones, and to achieve a difference between these groups, less tentative amounts of exercise should have been introduced to the training group. Another reason for the assumed lack of effect of our training program is owner compliance. Owners may have not followed the program as planned, thus affecting the outcome of the study. At the start of the study, we accepted a weaker than optimal study design by allowing the owners of the puppies to select the group (TRA or C) in which their dog would participate, instead of randomizing the dogs into groups. This was done to facilitate owner compliance, but at the same time this decision led to weak internal validity of our study. However, the study period was long (two-month training period) and required a high commitment from the owners, which, in some cases may have proven to be too much. This might also account for the finding of the step counts decreasing sooner than expected, i.e., prior to the end of training period and the start of the detraining period.

The recovery times after the sprints of both groups decreased similarly over time. This could be due to increased age and general fitness development, growth and physical maturation, or related to familiarization with the testing protocol. It can also reflect the puppies getting accustomed to the testing protocol and surroundings. It should be noted that in a previous study with Labrador retrievers, an abrupt increase in BL was not detected during a five-stage 30-minute incremental treadmill exercise test (35). Moreover, the decrease of BL during the 20-min recovery period was minimal (35). Although a change in BL was seen, it was not remarkable, and that might explain the minimal changes in our study as well. Again, whether this information is applicable to puppies is unknown, but it should be taken into account when assessing the appropriateness of our outcome measurement methods.

The outcome measures used in this study, i.e., the fitness field test and HR and lactate measurements, are new to puppies' exercise physiology. They provide abundant information and could be utilized in future studies on the topic. However, there were some unexpected practical challenges related to puppies' fitness testing, which have not been reported previously and which may have affected our results. Some of the puppies tried to bite the lead and play, some pulled on the lead, and some were easily distracted and stopped in the middle of their performance. Moreover, some puppies might urinate or defecate in the middle of the test. Thus, the 1,000 m run or the 200 m sprint might not have been a continuous and equal performance between all puppies. Some of these adversities could perhaps in future studies be avoided by using a treadmill-based test, rather than the field one that we employed. Furthermore, especially at the first testing time (baseline), the puppies were suspicious about the examinations and the attachment of the HR sensor belt. HRs at this point were often higher than otherwise. In later tests, some of the puppies also had higher HR before taking a blood sample for BL measurements due to them hearing the lactate sensor beep when it went on. All of these factors may have affected the fitness test results. However, the behavioral effect can be assumed to be the same in both groups, and thus, not to impact the group comparison results.

In practice, it is easiest to monitor the workload of endurance training by measuring HR during exercise (6). Heart rate monitors have previously been validated for use in dogs (36). The specific model is no longer manufactured, but an equivalent was used in our study. However, we encountered several issues with the use of the monitor. Keeping the HR-sensor belts in place during locomotion was challenging since for some of the puppies the belt slid constantly onto their abdomen or groin. Data for our study were collected over a one-year period, i.e., in summer as well as in winter. Particularly in the winter, puppies' thicker coat made it more difficult for the HR sensor to detect the HR. Shaving of the coat on the side of the HR-sensor could be helpful, but most of the owners did not allow this, especially in the winter. Moreover, both working Labradors and show Labradors were included in the study. With the working Labradors, sensors detected HR more easily: this might be due to the narrower chest conformation of working-line puppies.

Our hypothesis was that a training program would result in significantly lower HRs and BL concentrations and faster recovery times in the fitness test at all measurement timepoints. We aimed to provide growth-related reference values for HR and BL concentration. Our results do not fully support our hypothesis or our aim. Our study groups were small, and this surely impacted some of our results: for example, the lack of statistically significant differences between the groups cannot be interpreted as evidence of absence of a training effect. Thus, our findings should be considered preliminary ones and the study piloting by nature. Recruiting animals was not successful, mainly for COVID-19 pandemic-related reasons, and this limited the timeframe for performing data collection. The small sample size also exposes our results to type two bias. Hence, the fact that we could not show significant differences in outcome measurements between our groups might not mean that this is truly the case. Moreover, our C-group's values were intended to provide growth-related reference values, but due to the small sample size this aim was not achieved: the here reported values cannot be considered as reliable nor generalizable.

## Conclusion

Adaptation to training is always individual, and training should be periodized with more and less strenuous periods of varying volume, intensity, and frequency to reach optimal fitness development, as was seen in our study. However, the limited positive physiological changes of our exploratory study cannot be generalized due to the small number of animals and possibly due to excessive overall physical activity load leading to physiological changes not being measurable within the study period. Alternatively, an overly cautious training program may have resulted in the low training level in our study. We were unable to conclude the reasons for our limited results. Further exploration into the efficacy of the training program requires new studies with larger sample sizes and more strenuous, maximal HR-based exercise programs. Defining growth-related physiological reference values for young puppies also requires more information based on larger population samples.

## Data Availability

Due to its clinical nature, original data is not available to third parties. Requests to access the datasets should be directed to heli.hyytiainen@helsinki.fi.

## Footnotes

1 https://www.akc.org/most-popular-breeds/ (accessed October 13 2025).

2 https://www.kennelliitto.fi/koirat/suosituimmat-koirarodut/ (accessed October 13 2025).
