# Supplementary material for: Endurance training-induced changes in heart rate and blood lactate concentration in puppies
Source: Front Vet Sci. 2026 Mar 31;13:1770294. doi: 10.3389/fvets.2026.1770294 (PMC13076153; doi:10.3389/fvets.2026.1770294)
Supplement: Supplementary file 1 [file Table_1.docx]

Supplementary Material

**PUPPIES’ TRAINING PROGRAMME**

Once a week a day off, no lead exercising.

On the days, when there is physiotherapy / fitness test, no strenuous exercises.

**Week 1**

2 times a week physiotherapy

2 times a day 20 min lead walk brisk walking / pyppy jogging

Once a day 40 min brisk walking / puppy jogging

**Week 2**

2 times a week physiotherapy

2 times a day 30 min lead walk brisk walking / pyppy jogging

Once a day 50 min brisk walking / puppy jogging

**Week 3**

2 times a week physiotherapy

2 times a day 40 min lead walk brisk walking / pyppy jogging

Once a day 60min brisk walking / puppy jogging

**Week 4**

ONCE a week physiotherapy

ONCE a day 20 min lead walk brisk walking / pyppy jogging

Once a day 30 min brisk walking / puppy jogging

**Fitness test**

**Week 5**

2 times a week physiotherapy

2 times a day run 2 x 3 x 50m, recovery 2-3 min/6-8 min i.e.

i.e. run 50m fast 🡪 keep 2-3 min break 🡪 run the 50m again 🡪 2-3 min break 🡪 run again 🡪 6-8 min break, after which the three run sets are repeated

Once a day lead walk 40 min easy pace, although no sniffing and stopping

1 day off per week, no training or lead walking; puppy can play and move otherwise

**Week 6**

2 times a week physiotherapy

2 times a day run 2 x 2 x 100m, recovery 3-4 min / 8-10 min

Once a day lead walk 40 min easy pace

1 day off per week, no training or lead walk

**Week 7**

2 times week physiotherapy

2 times a day run 2 x 200m, recovery 10 min

Once a day lead walk 40 min easy pace

1 day off per week, no training or lead walk.

**Week 8**

2 times per week physiotherapy

Once a day lead walk 20 min

Once a day lead walk 40-50 min easy pace
